# Enhancement of antitumor immunotherapy using mitochondria-targeted cancer cell membrane-biomimetic MOF-mediated sonodynamic therapy and checkpoint blockade immunotherapy

**DOI:** 10.1186/s12951-022-01453-2

**Published:** 2022-05-14

**Authors:** Jiali Luo, Xue Wang, Zhan Shi, Yiqing Zeng, Liangcan He, Jing Cao, Yu Sun, Tao Zhang, Pintong Huang

**Affiliations:** 1 Department of Ultrasound in Medicine, The Second Affiliated Hospital of Zhejiang University School of Medicine, Zhejiang University, No. 88 Jiefang Road, Shangcheng District, Hangzhou, 310009 People’s Republic of China; 2 Research Center of Ultrasound in Medicine and Biomedical Engineering, The Second Affiliated Hospital of Zhejiang University School of Medicine, Zhejiang University, No. 88 Jiefang Road, Shangcheng District, Hangzhou, 310009 People’s Republic of China; 3 School of Medicine and Health, Harbin Institute of Technology, Harbin, 150080 People’s Republic of China

**Keywords:** Mitochondria-targeted, Cancer cell membrane, Metal–organic frameworks, R837, Immunotherapy

## Abstract

Immunotherapeutic interventions represent a promising approach to treating cancer, with strategies such as immune checkpoint blockade (ICB), immunogenic sonodynamic therapy (SDT), and immune adjuvant T cell delivery having exhibited clinical promise. In this report, we describe the use of cancer cell membrane-coated triphenylphosphonium (TPP) decorated nano-metal–organic framework (nMOF) constructs [Zr-TCPP(TPP)/R837@M] that were used to generate homologous, mitochondria-targeted platforms with a high rate of sonosensitizer loading. This construct was utilized to simultaneously promote tumor antigen presentation via enhancing SDT while synergistically promoting dendritic cell (DC) maturation through the delivery of the Toll-like receptor agonist R837. In vitro, these functionalized nMOFs were readily internalized by homologous tumor cells in which they were efficiently targeted to the mitochondria, promoting DC activation through the induction of immunogenic cell death (ICD) following ultrasound exposure. Moreover, this nanoplatform was able to achieve in vivo synergy with anti-CTLA-4 ICB to reverse immunosuppression tumor microenvironment (TME), thus achieving more robust antitumor efficacy capable of suppressing metastatic disease progression and facilitating the development of durable antitumor memory responses. Together, these results highlight a promising approach to achieving enhanced SDT activity while overcoming an immunosuppressive TME, thereby achieving more robust antitumor immunity.

**Supplementary Information:**

The online version contains supplementary material available at 10.1186/s12951-022-01453-2.

Cancer immunotherapy strategies leverage host immune responses to target and eliminate malignant neoplasms, and several such approaches have emerged as promising antitumor treatments in recent years [1–5]. Immune checkpoint blockade (ICB) strategies targeting immunosuppressive proteins including cytotoxic T-lymphocyte-associated antigen 4 (CTLA-4) have been shown to achieve marked clinical efficacy in a range of tumor types [6–8]. In addition to overcoming immunosuppression, the establishment of a robust antitumor immune response is dependent on tumor-associated antigen (TAA) release and the subsequent presentation of these antigens by dendritic cells (DCs) and other antigen-presenting cells (APCs) to T cells [9, 10]. ICB is thought to fail in many treated patients owing to a dearth of TAAs within the tumor microenvironment (TME) and insufficient DC-mediated antigen presentation.

Sonodynamic therapy (SDT) has emerged as a promising approach to enhancing antigen presentation [11, 12]. SDT is an inexpensive and nonionizing therapeutic modality that is easily controlled and can effectively penetrate target tissues, killing cancer cells by generating highly cytotoxic singlet oxygen (^1^O_2_) [13–15]. Importantly, SDT promotes highly immunogenic cell death (ICD) and the associated release of TAAs and other tumor debris, thus enhancing DC recruitment, activation, and T cell priming [16–18]. The efficacy of SDT strategies is thus closely tied to the consequent induction of ICD and associated TAA release. Antigen presentation can also be augmented by stimulating DCs with Toll-like receptor (TLR) agonists [19], including imiquimod (R837), which binds to TLR7 and promotes DC maturation [20, 21]. Despite its immunogenic potential, R837 exhibits poor pharmacokinetic properties and very limited water solubility, constraining its ability to effectively activate DCs in vivo [22, 23].

Nano-metal–organic frameworks (nMOFs) are polymers formed through the coordination of organic ligands with metal ions or clusters [24, 25]. These nMOFs are highly porous, exhibit a large surface area, and are highly tunable with respect to pore size and surface chemistry, enabling them to avoid toxic biodegradation. As such, they have been explored as promising drug carriers capable of use in nano vaccine platforms for the delivery of immunostimulatory adjuvants and TAAs capable of activating DCs and promoting more robust immunotherapy outcomes [26–28]. By modifying these nMOFs with a triphenylphosphonium (TPP) vector, they can be more reliably targeted to the mitochondria within target cells, which is an attractive possibility owing to the central role played by these organelles in the regulation of ATP generation, reactive oxygen species (ROS) production, and apoptotic cell death [29–31]. By targeting the mitochondria, it may be possible to selectively enhance SDT efficacy. Porphyrin-based nMOFs can also serve as reliable sonosensitizers (SNSs), further improving the efficiency of SDT treatment [32–34]. To avoid mononuclear phagocyte-mediated clearance of systemically administered nanomaterials that can compromise their intratumoral accumulation and circulation, biomimetic tumor cell membranes can be employed [35–38]. These membranes enable nanomaterials to more effectively evade immune-mediated detection, thereby prolonging the circulation of encapsulated drugs. By combining mitochondria-targeted porphyrin-based nMOFs with tumor cell membranes, it can be called a cascade bioreactor, which is possible to retain the efficient drug delivery properties and bioactivity of nMOFs while achieving more reliable tumor targeting and anticancer activity.

In this study, we synthesized TPP-conjugated porphyrin-based nMOFs (Zr-TCPP(TPP)/R837@M) modified with 4T1 cell membranes to achieve homologous mitochondria-targeted SDT efficacy in combination with simultaneous delivery of R837 as an immune adjuvant. Moreover, we successfully combined this enhanced SDT immunotherapeutic platform with anti-CTLA-4, leveraging the high SNS loading rate of the MOF structure together with a robust sequential targeting mechanism. The SDT-induced in situ release of TAAs derived from primary tumors can elicit vaccine-like activity, particularly in combination with an immune adjuvant, eliciting robust immunity by inducing the maturation of DCs and associated cytokine secretion and tumor killing. When combined with anti-CTLA-4 ICB treatment, such antitumor immunity can be further enhanced, as evidenced by increases in intratumoral CD8^+^ T cell infiltration. This synergistic immunotherapy platform was able to suppress primary tumor growth and metastasis in orthotopic, subcutaneous, and artificial systemic metastasis murine 4T1 breast tumor model systems (Scheme 1). This combination immunotherapy approach was also conducive to the development of durable immunological memory, protecting against tumor rechallenge following primary tumor elimination. Overall, this study highlights the promise of utilizing sequential targeted SDT together with ICB to treat tumors.

**Scheme 1:**
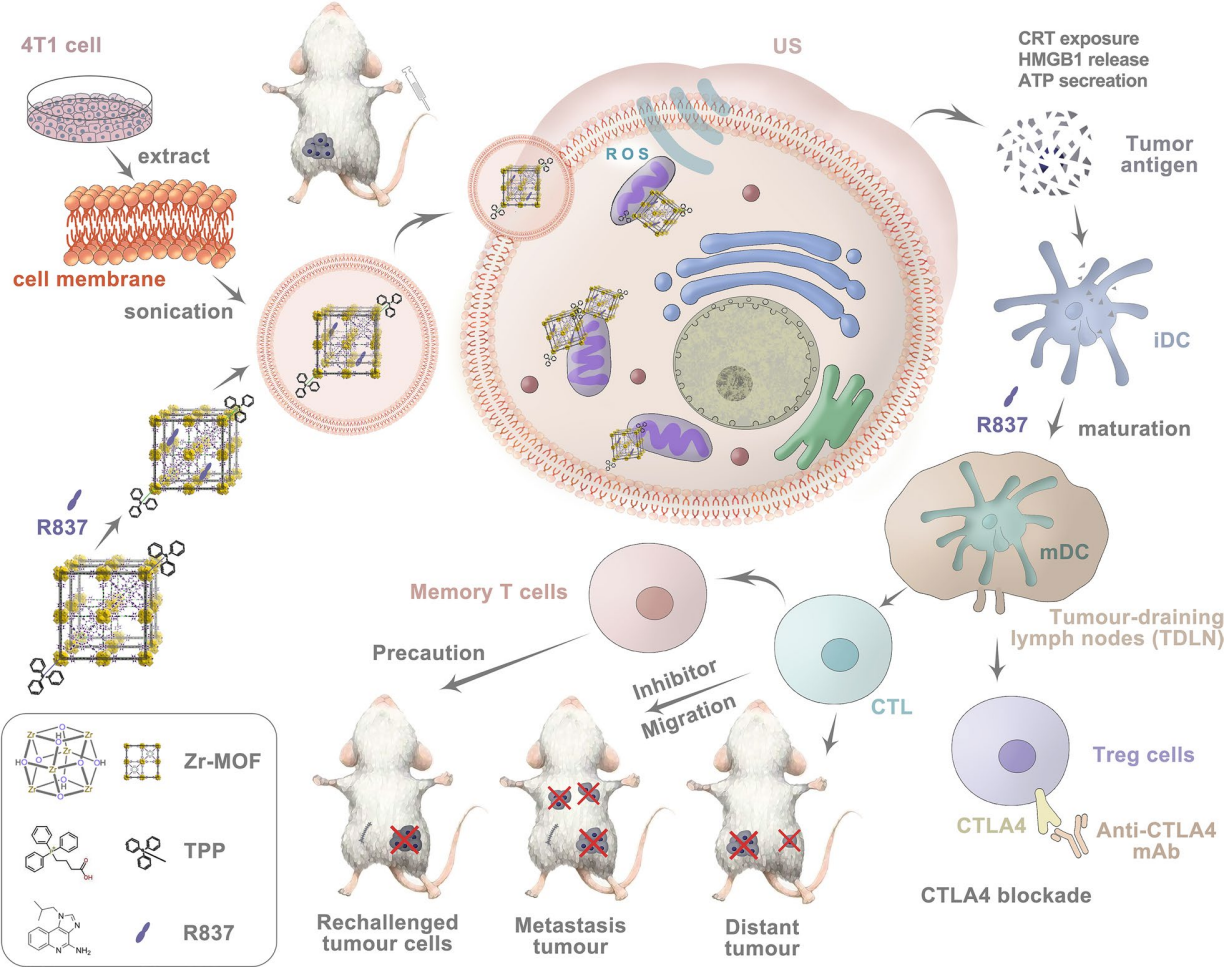
Schematic illustration mechanism of mitochondria-targeted cancer cell membrane-biomimetic MOF-mediated SDT and immune checkpoint blockade immunotherapy

## Results and discussion

**Fig. 1:**
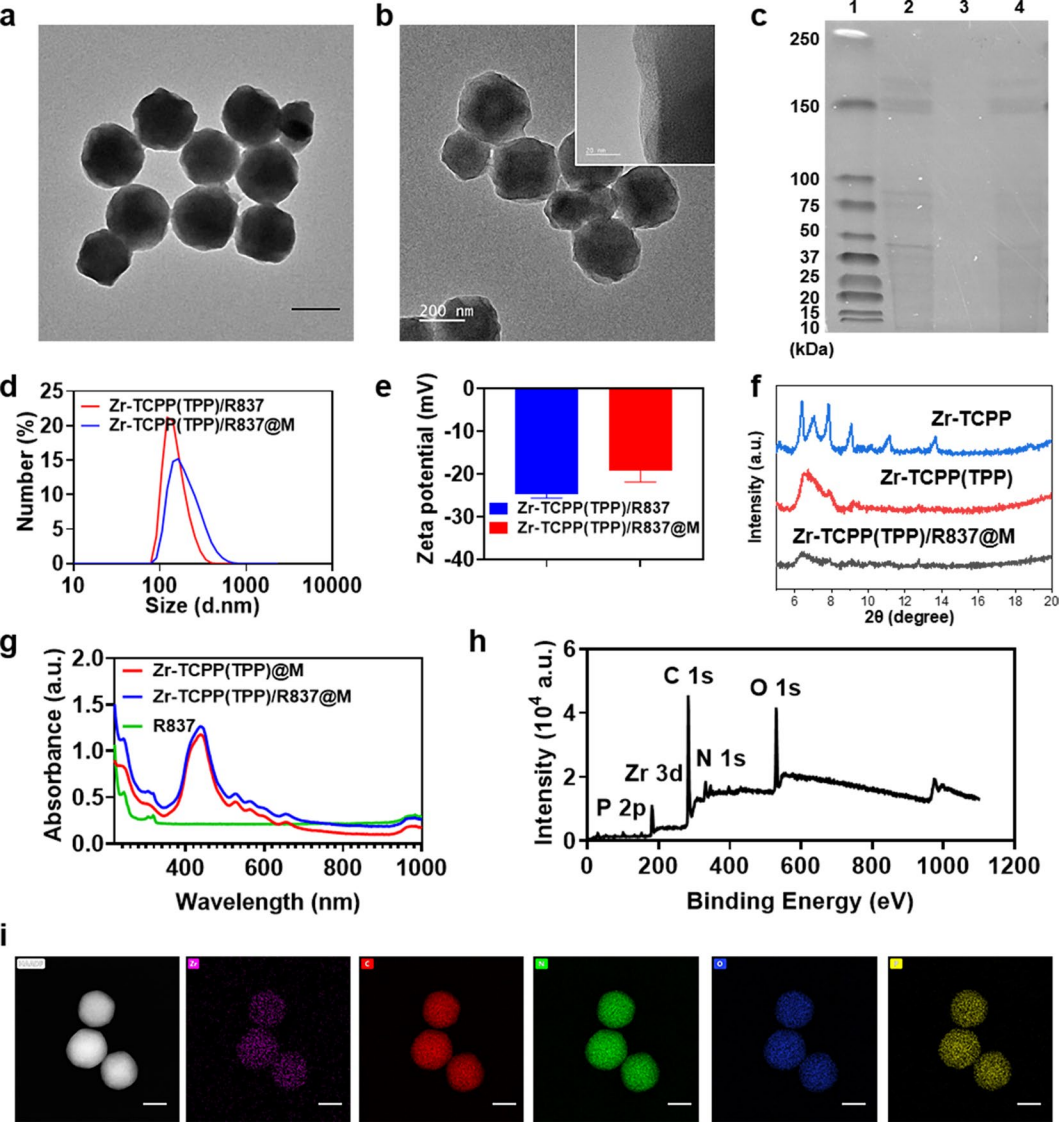
Charaterisations of the nanosonosensitisers. **a** TEM image and magnified TEM Image of Zr-TCPP(TPP)/R837@M nanoparticles, scale bar: 200 nm. **b** High-resolution TEM assessment of Zr-TCPP(TPP)/R837@M. **c** SDS-PAGE protein analysis: L1, marker; L2, 4 T-1 cell membrane(M); L3, Zr-TCPP(TPP)/R837; L4, Zr-TCPP(TPP)/R837@M. **d** Hydrodynamic size distribution of Zr-TCPP(TPP)/R837 and Zr-TCPP(TPP)/R837@M. **e** Surface ζ potential of Zr-TCPP(TPP)/R837 and Zr-TCPP(TPP)/R837@M (n = 3) **f** the power XRD spectrum of Zr-TCPP nanoparticles. **g** UV–vis spectrum of R837, Zr-TCPP(TPP)@M and Zr-TCPP(TPP)/R837@M. **h** XPS spectra of Zr-TCPP(TPP)/R837@M. **i** The elements mapping of Zr-TCPP(TPP) of Zr, C, N, O, P, scale bar: 100 nm

R837-loaded Zr-TCPP(TPP) NPs were prepared and coated with 4T1 cell-derived membranes (M) to generate Zr-TCPP(TPP)/R837@M particles via extrusion (Scheme 1). Transmission electron microscopy (TEM) revealed these Zr-TCPP(TPP)/R837@M to be spherical in shape with the expected core–shell structure (Fig. 1a). High-resolution TEM (HR-TEM) further confirmed the presence of cell membranes surrounding Zr-TCPP(TPP)/R837 particles (Fig. 1b), while SDS-PAGE analyses demonstrated that 4T1 cell membrane-derived proteins were detectable within these Zr-TCPP(TPP)/R837@M preparations (Fig. 1c). The average hydrodynamic diameter of the Zr-TCPP(TPP)/R837@M particles in dynamic light scattering (DLS) analyses was 216.30 ± 8.78 nm, and they exhibited a polydispersity index (PDI) of 0.191 (Fig. 1d). The zeta potential of Zr-TCPP(TPP)/R837@M rose to − 19.23 ± 5.1 mV following extrusion, consistent with successful membrane coating (Fig. 1e). In the X-ray diffraction (XRD) spectrum, major Zr-TCPP(TPP)/R837 peaks were consistent with those of simulated and actual Zr-TCPP preparations, indicating that Zr-TCPP(TPP)/R837 samples harbor the same framework as Zr-TCPP (Fig. 1f).

In the Zr-TCPP(TPP)/R837@M UV–vis absorption spectrum, a characteristic peak at ~ 320 nm assigned to R837 in the UV–vis absorption spectrum is consistent with successful R837 encapsulation in these nMOFs (Fig. 1g). Zr-TCPP(TPP) XPS spectra indicated that O, N, C, Zr, and P were present within prepared samples (Fig. 1h). We further confirmed the elements distribution of Zr-TCPP(TPP) via STEM-based elemental mapping (Fig. 1i), with P and Zr respectively corresponding to TPP and Zr-MOF, confirming the connection of TPP.

Next, NP uptake by 4T1 cells was assessed in vitro by combining these cells with Zr-TCPP(TPP)/R837@M NPs for a range of time periods (0, 1, 4, 8, 12, and 24 h) and then assessing cells via flow cytometry. Within a 12 h incubation period, substantial Zr-TCPP(TPP)/R837@M NP uptake was detectable within 4T1 cells (Additional file 1: Figure S1a). When a range of Zr-TCPP(TPP)/R837@M concentrations (up to 200 µg/mL) were used to treat 4T1 cells for 24 h, these NPs were found to induce only limited cytotoxicity (Additional file 1: Figure S1b).

**Fig. 2:**
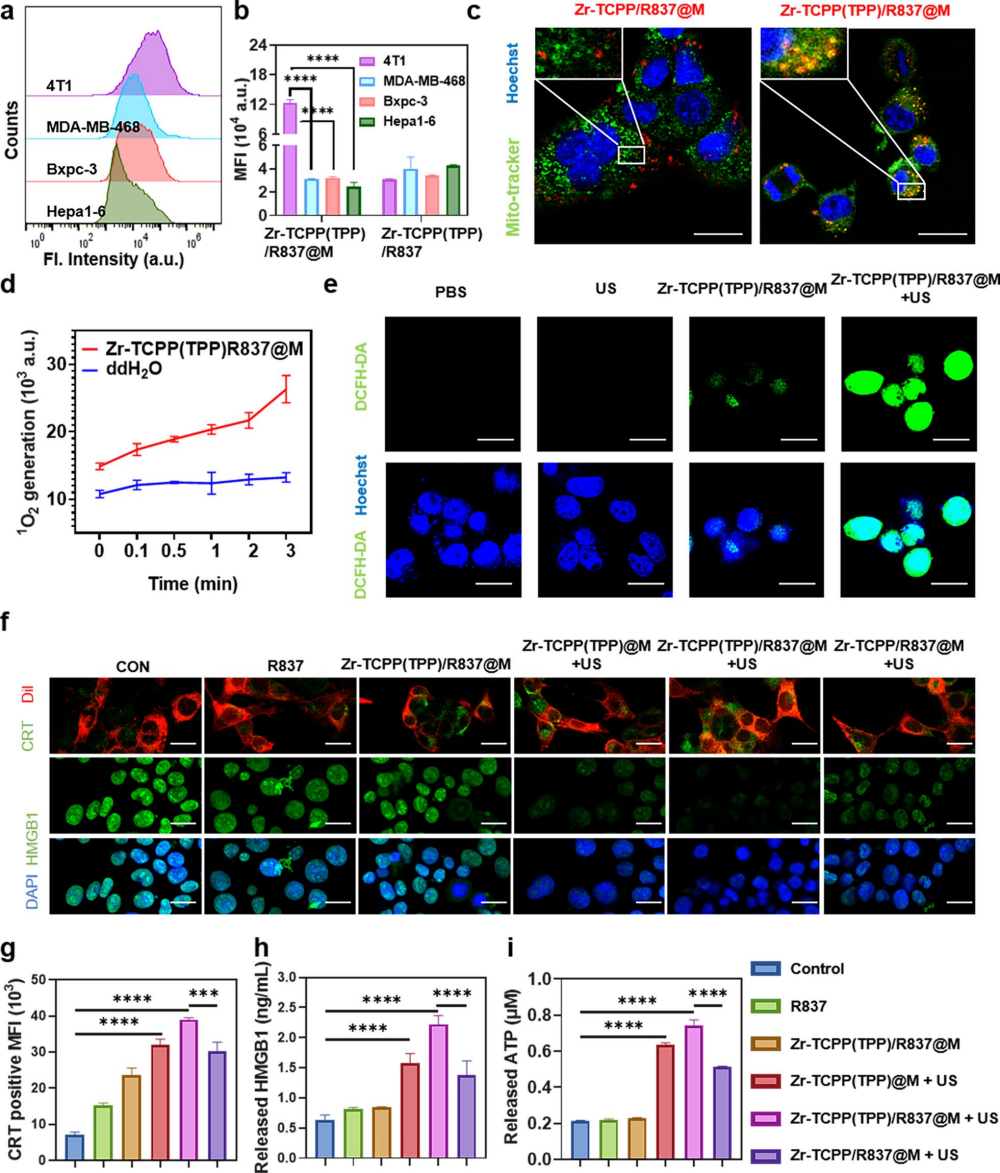
In vitro uptake and stimulation of in vitro immune response. **a** Homotypic effect of Zr-TCPP(TPP)/R837@M on four cell lines, namely 4T1, MDA-MB-468, Hepa1-6, and Bxpc-3, examined by flow cytometry. **b** Statistical analysis of four cell lines after 8 h incubation with Zr-TCPP(TPP)/R837@M or Zr-TCPP(TPP)/R837. **c** CLSM images to show subcellular localization of Zr-TCPP(TPP)/R837@M, and Zr-TCPP/R837@M. Scale bar: 25 μm. **d** Time related SOSG (5 μM) fluorescence changes of Zr-TCPP(TPP)/R837@M under US irradiation. **e** CLSM images of 4T1 cells after treated with of Zr-TCPP(TPP)/R837@M with or without US irradiation. DCFH-DA (10 μM) was employed as the intracellular ROS sensor. Scale bar: 25 μm. **f** CRT exposure and HMGB1 release in 4T1 cells treated with different formulations, following by CLSM. Scale bar: 25 μm. **g** Flow cytometry analysis of CRT exposure in different groups. **h** HMGB1 release in cell supernatant after various treatments. **i** ATP secretion in different groups. Data are expressed as means ± SD (n = 3). Statistical significances were calculated via one-way ANOVA Tukey's multiple comparisons test, *P < 0.05, **P < 0.01, ***P < 0.001

To assess the homologous targeting activity of Zr-TCPP(TPP)/R837@M, these NPs were incubated with 4T1 murine breast cancer cells or with other tumor cell lines including the MDA-MB-468 human breast cancer cell line, the Hepa1-6 murine hepatocellular carcinoma cell line, and the Bxpc-3 human pancreatic cancer cell line. Of these four cell lines, Zr-TCPP(TPP)/R837@M uptake was significantly higher in 4T1 cells, while the uptake of Zr-TCPP(TPP)/R837 in 4T1 cells didn’t show any increase compared with other cell lines, confirming the specificity with which Zr-TCPP(TPP)/R837@M particles can bind to homologous 4T1 cells (Fig. 2a, b; Additional file 1: Figure S2). The prepared Zr-TCPP(TPP)/R837@M samples were then utilized to facilitate mitochondrial-targeted SDT in target 4T1 cells. Relative to control treatment, Zr-TCPP/R837@M was found to efficiently enter into these tumor cells even in the absence of specific mitochondria targeting (Fig. 2c), with a mitochondrial colocalization coefficient Pearson’s R value (no threshold): 0.17. In contrast, Zr-TCPP(TPP)/R837@M was both efficiently internalized by these 4T1 cells and preferentially accumulated in the mitochondria, with a mitochondrial colocalization coefficient Pearson's R value (no threshold): 0.62.

To evaluate Zr-TCPP(TPP)/R837@M-induced ROS yields, a singlet oxygen sensor green (SOSG; 1 μM) reagent was selected to trap and detect ^1^O_2_. With the prolongation of US exposure time, SOSG fluorescent intensity rose substantially (Fig. 2d). DCFH-DA was then chosen as a fluorescent indicator to gauge intracellular ROS levels. After Zr-TCPP(TPP)/R837@M + US exposure and irradiation for 1 min, a bright green fluorescent signal was evident in these cells, while this signal was less robust in cells from the US or Zr-TCPP(TPP)/R837@M treatment groups (Fig. 2e).

We additionally examined the impact of Zr-TCPP(TPP)/R837@M-based SDT on the induction of ICD by quantifying calreticulin (CRT) exposure, the release of high mobility group box 1 (HMGB1), and the secretion of ATP. CLSM imaging revealed Zr-TCPP(TPP)/R837@M to effectively promote the exposure of CRT on 4T1 cell surfaces when Dil was used for membrane staining (Fig. 2f). The release of HMGB1 from the nuclei of these cells was apparent in the Zr-TCPP(TPP)@M, Zr-TCPP(TPP)/R837@M and Zr-TCPP(TPP)@M groups within 1 min following US exposure, especially in the Zr-TCPP(TPP)/R837@M + US group, which almost invisible fluorescence. The result of HMGB1 was also confirmed by the ELISA test (Fig. 2h). Flow cytometry analyses further demonstrated that Zr-TCPP(TPP)/R837@M-treated cell exposure to US induced CRT exposure to an extent greater than that observed in other treatment groups (Fig. 2g; Additional file 1: Figure S3). Owing to the ROS-mediated induction of mitochondrial damage, Zr-TCPP(TPP)/R837@M + US treatment was associated with significantly increased ATP secretion as compared to other treatments (Fig. 2i). In conclusion, these data confirmed that Zr-TCPP(TPP)/R837@M-based SDT was able to effectively induce in vitro tumor cell ICD.

**Fig. 3:**
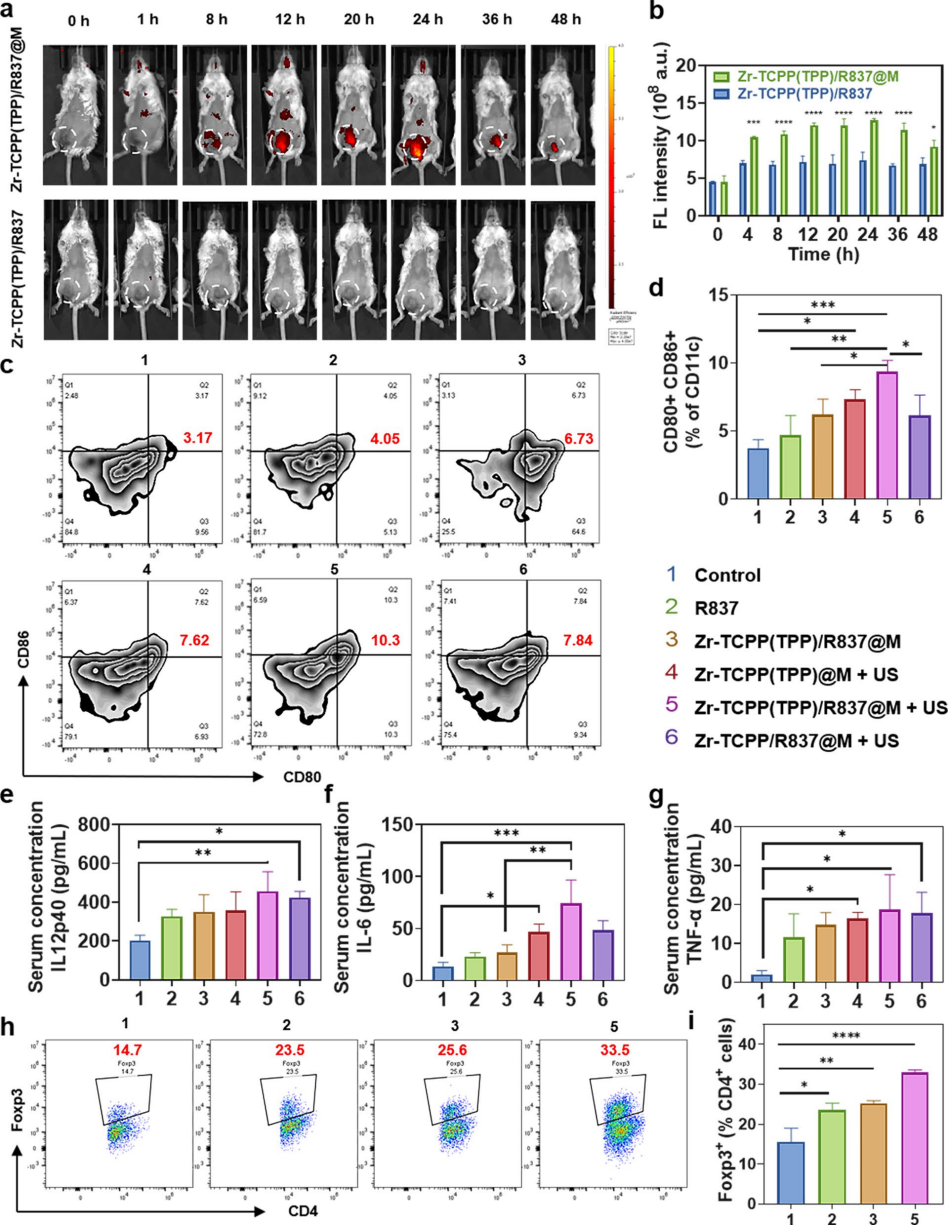
In vivo evaluation of tumor targeting and immune responses. **a** In vivo fluorescence images to reveal the biodistribution of Zr-TCPP(TPP)/R837 and Zr-TCPP(TPP)/R837@M post i.v. injection into 4T1-tumour-bearing mice at the indicated time points. White circles indicate tumours; **b** the accumulation curve of Zr-TCPP(TPP)/R837 and Zr-TCPP(TPP)/R837@M in the tumour tissue by measuring the fluorescence intensity of tumours at different time points post i.v. injection, error bars are based on SD (n = 3); **c**, **d** DC maturation in the tumour-draining lymph nodes induced by various treatments on mice bearing 4T1 tumours, as assessed by flow cytometry after staining with CD11c, CD80, CD86 and live dead; **e**–**g** cytokine levels of TNF-α, IL-6 and IL-12p70 in sera from mice after different treatment. **h**, **i** Flow cytometry plots and the corresponding quantification showing percentages (gated on CD4^+^ cells) of CD4^+^Foxp3^+^ Treg cells in tumours tumors after various treatments indicated. Data are expressed as means ± SD (n = 3). Statistical significances were calculated via one-way ANOVA Tukey's multiple comparisons test, *P < 0.05, **P < 0.01, ***P < 0.001

When Zr-TCPP(TPP)/R837 and Zr-TCPP(TPP)/R837@M biodistributions were assessed in BALB/c mice bearing 4T1 tumors using an IVIS system, Zr-TCPP(TPP)/R837@M accumulation within tumors was evident within 24 h of treatment followed by gradual reductions in these levels over time, whereas Zr-TCPP(TPP)/R837 exhibited reduced levels within these 4T1 tumors at all tested time points (Fig. 3a, b). Ex vivo fluorescent analyses indicated that Zr-TCPP(TPP)/R837 and Zr-TCPP(TPP)/R837@M were readily metabolized within 24 h following injection in most assessed organs, with Zr-TCPP(TPP)/R837@M treatment being associated with higher fluorescence intensity values as compared to Zr-TCPP(TPP)/R837 treatment (Additional file 1: Figure S4a, b). Together, these results suggested that Zr-TCPP(TPP)/R837@M was able to more readily accumulate within homologous tumor cells owing to the tumor-targeting activity of the 4T1 cell membranes used for NP encapsulation.

Several different therapeutic modalities including photodynamic therapy, photothermal therapy, and SDT have been shown to promote antitumor immunity by inducing ICD and consequent TAA release. These TAAs, in turn, can function in a vaccine-like manner when an immune adjuvant induces DC maturation and consequent cytokine production and cytolytic cell killing. SDT treatment in the Zr-TCPP(TPP)/R837@M + US treatment group was associated with clear increases in lymph node DC maturation (9.36 ± 0.82%) to levels above those in other groups including the Zr-TCPP(TPP)/R837@M only group (6.19 ± 1.16%) or the US + Zr-TCPP(TPP)@M (7.33 ± 0.71%) treatment group in which R837 was absent. Moreover, R837 alone induced only very low levels of DC maturation (4.67 ± 1.48%). Zr-TCPP(TPP)/R837@M + US treatment also induced more robust DC maturation than did non-mitochondria-targeting Zr-TCPP/R837@M + US treatment (6.15 ± 1.49%), consistent with the beneficial effects of mitochondria targeting (Fig. 3c, d). In line with the results of DC maturation assays, tumor cell debris release following Zr-TCPP(TPP)/R837@M-enhanced SDT treatment was sufficient to augment the production of key immune-related cytokines including IL-6, IL-12p40, and TNF-α by DCs, further confirming the ability of R837 to function as an immune adjuvant in this assay context, enhancing overall immune response induction (Fig. 3e–g). While IL-6 and IL-12 are important mediators of the activation of innate immune NK cells, TNF-α plays a central role in the induction of adaptive cell-mediated immunity necessary for a robust tumor immunotherapy response. As such, TAAs released from tumor cell debris following SDT, together with immunostimulatory adjuvants, were able to effectively trigger the maturation of DCs.

While eliciting ICD can benefit antitumor immunity, intratumoral feedback mechanisms can suppress immune activation and thereby compromise the effective induction of a robust or durable immune response [39, 40]. Regulatory T cells (Tregs) are frequently present at high levels within tumors wherein they function to suppress immune responses and associated cytolytic tumor cell killing [41, 42]. Flow cytometry analyses revealed clear increases in the levels of Tregs (CD4^+^ Foxp3^+^) following Zr-TCPP(TPP)/R837@M-enhanced SDT treatment (Fig. 3h, i). As such, we posit that the antitumor immune responses that were induced as a result of SDT-induced ICD may, in the absence of other treatments, be suppressed by the post-SDT recruitment of Tregs into the TME.

**Fig. 4:**
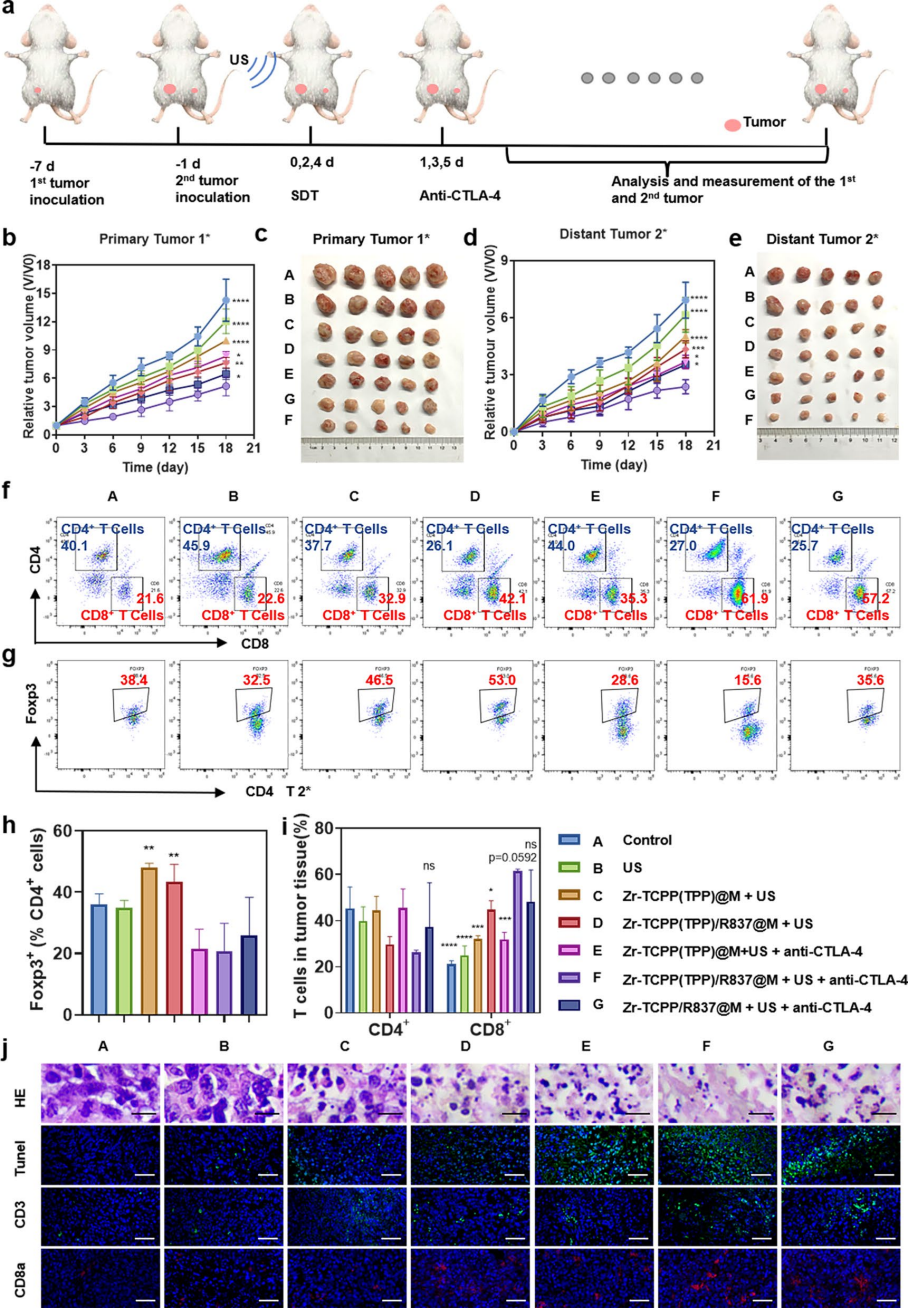
Antitumor immune effects of US in combination with checkpoint blockade. **a** Schematic illustration of our experiment design. Mice with 4T1 tumors on both sides were used. Tumors on the right side were designated as “primary tumors” for US, and those on the left side were designated as “distant tumors” without direct exposure to US. Growth curves and excised tumors at the end of treatments for **b**, **c** primary tumors and **d**, **e** distant tumors on mice after various treatments indicated. (n = 5). Statistical significances were calculated via one-way ANOVA Tukey's multiple comparisons test, *P < 0.05, **P < 0.01, ***P < 0.001. **f**, **g** Representative flow cytometry plots showing different types of T cells in secondary tumors from different groups of mice, and **h**, **i** proportions of tumor-infiltrating CD4 + and CD8 + T cells among all cancer cells. Proportions of Foxp3 + Tregs among CD4 + T cells. (n = 3) Statistical significance was calculated by one-way ANOVA Dunnett's multiple comparisons test. *P*-value: *, *P* < 0.05;**, *P* < 0.01; ***, *P* < 0.001. **j** In vivo apoptosis and/or necrosis of the tumour induced by different treatment, as shown by H&E staining and TUNEL assay; representative CLSM images of the mimic distant tumours after immunofluorescence staining (scale bar = 50 μm)

The antibody-mediated blockade of the immunosuppressive immune checkpoint protein CTLA-4 can suppress Treg activity. In addition, simultaneous CTLA-4 ICB in combination with other antitumor treatments can readily induce tumor-specific immune responses, thereby eliminating residual tumor cells that may be present at the site of tumor metastasis. We next evaluated the therapeutic efficacy of combining Zr-TCPP(TPP)/R837@M-enhanced SDT with anti-CTLA-4 treatment. For these analyses, primary 4T1 tumors were implanted on the right side of each mouse, with an artificial metastatic tumor then being established 1 week later on the left side of these same mice (Fig. 4a). Tumor growth in these animals was then monitored to evaluate treatment outcomes in primary and secondary tumors (Fig. 4b–e; Additional file 1: Figure S5a). US treatment in the absence of other therapeutic interventions failed to inhibit the growth of primary or distant tumors. While Zr-TCPP(TPP)/R837@M-enhanced SDT treatment was sufficient to inhibit primary tumor growth, it failed to impact the growth of metastasis-mimicking distant tumors, and its antitumor activity was enhanced in the context of anti-CTLA-4 combination treatment. Importantly, Zr-TCPP/R837@M-augmented SDT together with anti-CTLA-4 blockade was sufficient to eradicate primary 4T1 tumors (volume reduction rate, VRR, 59.5%) while also markedly suppressing distant tumor growth (VRR, 61.4%). Consistent with the above results, Zr-TCPP(TPP)/R837@M-enhanced SDT + anti-CTLA-4 combination treatment was more readily able to inhibit tumor growth than was an equivalent therapeutic strategy lacking mitochondria targeting activity, consistent with the therapeutic benefits of this intervention. When assessing the biosafety and toxicology of Zr-TCPP(TPP)/R837@M treatment in vivo*,* the hemolysis rate was found to be < 5% following red blood cell treatment with a range of Zr-TCPP(TPP)/R837@M concentrations (Additional file 1: Figure S6). These NPs are thus highly biocompatible and unlikely to induce systemic toxicity. Consistently, no abnormalities or organ damage were visible upon the hematoxylin and eosin (H&E) staining major organs (heart, lungs, liver, kidneys, and spleen) of treated mice (Additional file 1: Figure S7).

To explore the mechanisms whereby Zr-TCPP(TPP)/R837@M-enhanced SDT + anti-CTLA-4 treatment achieves robust therapeutic efficacy, distant tumors were collected from mice on day 7 post-treatment and assessed via flow cytometry (Fig. 4f–i). The frequency of intratumoral tumor-infiltrating lymphocytes (TILs, CD3^+^) rose following Zr-TCPP(TPP)/R837@M-enhanced SDT + anti-CTLA-4 treatment, with a particularly marked increase in intratumoral CD8^+^ T cell infiltration (Additional file 1: Figure S5b). The absolute CD8^+^ T cell frequency in the combination treatment group was 61.53 ± 0.81%, which was almost 2.9-fold higher than that in the PBS treatment group (21.10 ± 1.61%), and was also significantly higher than that observed in the group without mitochondrial targeting (48.27 ± 13.65%, *P* = 0.0592) (Fig. 4f, h). Together, these results indicated that Zr-TCPP(TPP)/R837@M-enhanced SDT + anti-CTLA-4 treatment promotes the immunosuppressive TME more conducive to the development of a productive immune response owing to the recruitment of many immune cells to target tumors.

Assessing the presence of Foxp3^+^ Tregs within human breast tumors can offer valuable insight regarding disease progression and prognosis, making these cells a valuable target for therapeutic intervention owing to their ability to suppress T cell proliferation and effector cytokine production [43]. When leukocytes were collected from distant tumors in our assay system and examined for CD3, CD4, and Foxp3 expression, no significant changes in CD4^+^T cell frequencies were observed in distant tumors, whereas the frequency of immunosuppressive Tregs declined in these distant tumors following Zr-TCPP(TPP)/R837@M-enhanced SDT treatment, suggesting that anti-CTLA-4 treatment effectively suppressed the activity of Tregs recruited into these distant tumors following primary tumor SDT treatment (Fig. 4g–i).

Histological and immunofluorescent staining of tumor tissue sections was next conducted (Fig. 4j). TUNEL staining indicated that the greatest levels of apoptotic and necrotic cell death were evident in the Zr-TCPP(TPP)/R837@M-enhanced SDT + anti-CTLA-4 treatment group, consistent with the superior combinatory efficacy of this therapeutic modality. Immunofluorescent staining demonstrated that this combined therapeutic approach enhanced CD3^+^T cell infiltration into distant tumors, whereas such infiltration was largely absent in animals subjected to PBS treatment. The majority of these tumor-infiltrating T cells were CD8^+^, further confirming the ability of Zr-TCPP(TPP)/R837@M-enhanced SDT + anti-CTLA-4 treatment to markedly enhance intratumoral CD8^+^T cell infiltration.

**Fig. 5:**
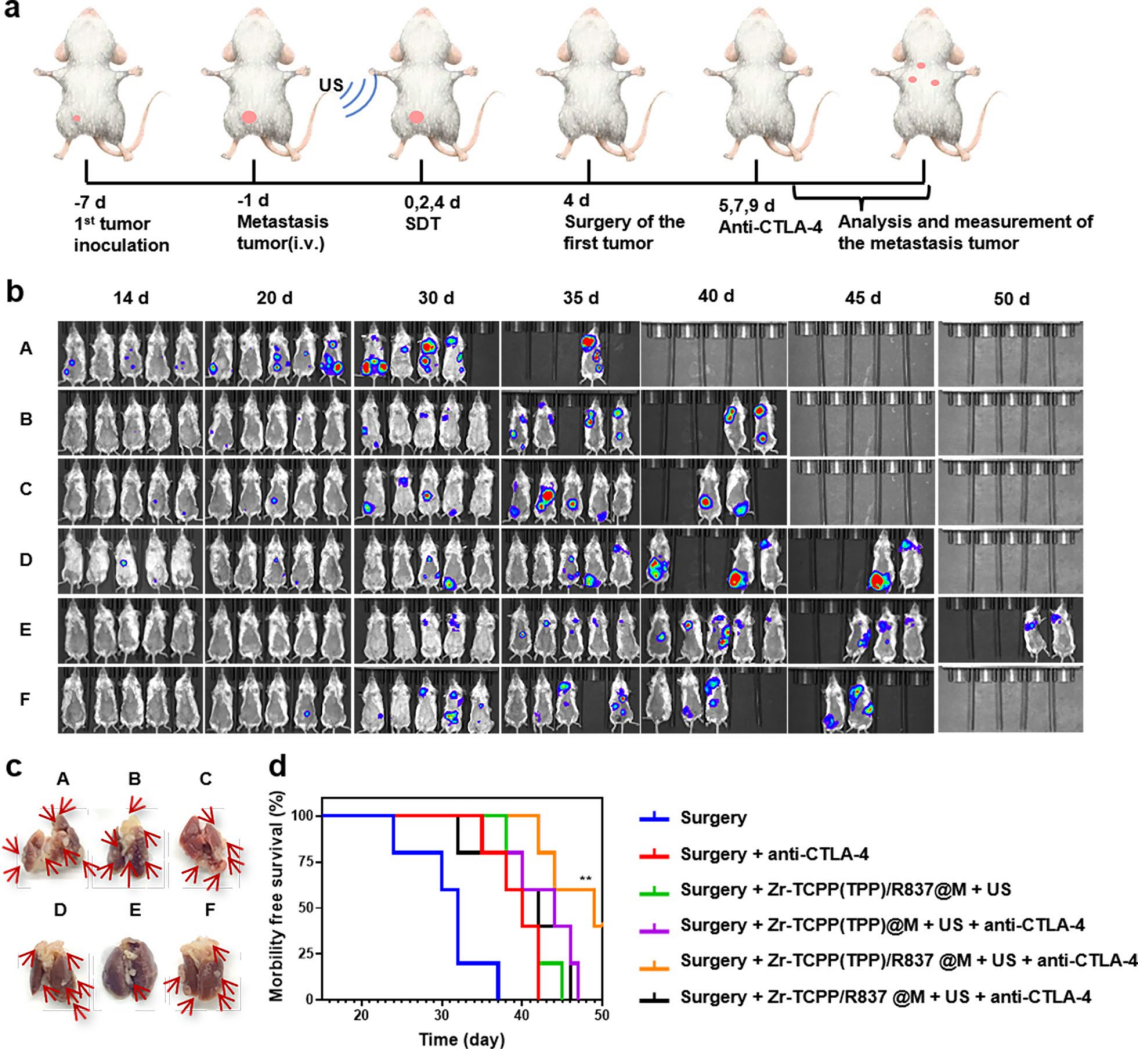
Tumor metastasis inhibition by SDT with Zr-TCPP(TPP)/R837@M combined anti-CTLA-4 therapy on the 4T1 orthotopic breast tumor metastasis model. **a** Schematic illustration of our experimental design by using Zr-TCPP(TPP)/R837@M-based SDT combined anti-CTLA-4 therapy to inhibit orthotopic cancer metastasis. FLuc-4T1 cells were inoculated into the breast pad of each mouse to establish the orthotopic murine breast cancer. Six days later, fLuc-4T1 cells were i.v. injected into each mouse to trigger whole-body spreading of cancer cells. **b** In vivo bioluminescence images to track the spreading and growth of fLuc-4T1 cancer cells in different groups of mice after various treatments to eliminate their primary orthotopic tumors. **c** Representative photographs to show the gross appearance of tumour nodules in the lungs. **d** Morbidity free survival of different groups of mice with metastatic 4T1 tumors after various treatments indicated to eliminate their primary orthotopic tumors (n = 5). Statistical significance was calculated by Log-rank (Mantel-Cox) test. *P*-value: *, *P* < 0.05;**, *P* < 0.01

Given the promising efficacy observed in our orthotopic tumor model system, we additionally examined the ability of this combination therapeutic regimen to achieve therapeutic benefit in a model that more closely mimics aggressive metastatic tumor growth. Tumor growth and metastasis were monitored via in vivo bioluminescent imaging in different treatment groups (Fig. 5a). For mice in which primary breast tumors had been surgically removed, a clear bioluminescent signal consistent with tumor metastasis was evident on day 14 following intravenous fLuc-4T1 cell injection, even in animals that underwent anti-CTLA-4 treatment or combination Zr-TCPP(TPP)/R837@M-augmented SDT (Fig. 5b). When animals were instead subjected to US treatment following injection with nMOFs lacking mitochondrial targeting activity together with anti-CTLA-4 treatment, only a slight delay of metastatic progression was evident, while Zr-TCPP(TPP)/R837@M-augmented SDT + anti-CTLA-4 strongly suppressed such metastasis (Fig. 5b). When dissected lung nodules were assessed in these mice, this analysis further confirmed the therapeutic benefits of combination therapy (Fig. 5c). When murine survival following treatment was monitored (n = 5 mice/group), 40% of mice in the combination Zr-TCPP(TPP)/R837 @M-enhanced SDT + anti-CTLA-4 treatment group survived for 50 days, whereas no mice in the other groups (Fig. 5d). The data further indicate that combining SDT, Zr-TCPP(TPP)/R837@M, and CTLA-4 ICB can effectively induce systemic antitumor immunity conducive to preventing tumor metastasis and prolonging survival in a murine model of metastatic disease.

**Fig. 6:**
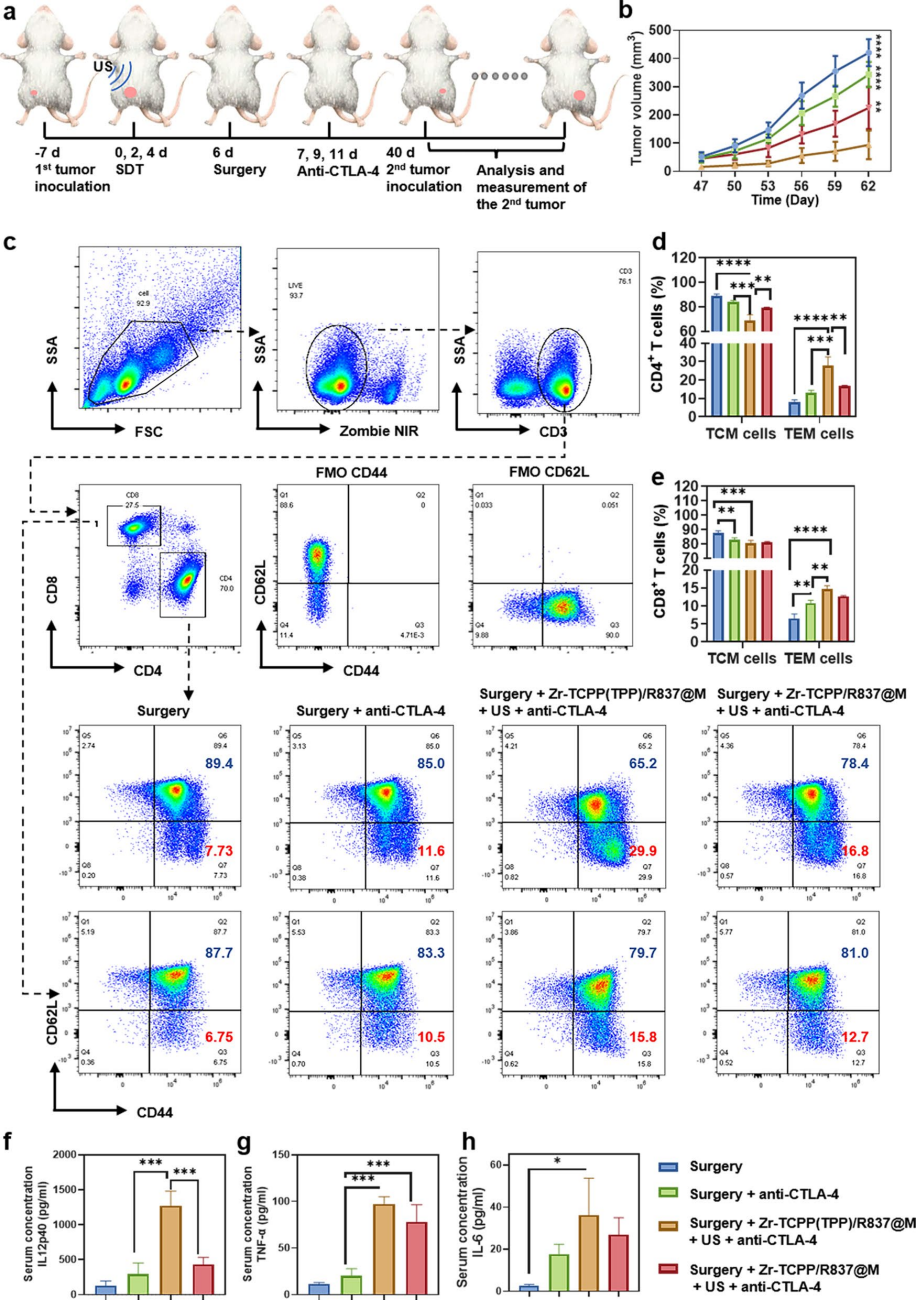
Long-term immune-memory effects induced by Zr-TCPP(TPP)/R837@M-based SDT. **a** Schematic illustration of the experiment design to assess the anti-tumour immune responses against mimic distant tumours and the immunological memory response triggered by Zr-TCPP(TPP)/R837@M -augmented SDT and anti-CTLA-4 combination to inhibit cancer relapse. **b** Tumor growth curves of rechallenged tumors inoculated 40 days post eliminated of their first tumors (n = 5) by different treatment. **c** Representative flow-cytometry plots of Lymph nodes cells of 4T1 tumour-bearing mice treated with the combined immunotherapy at day 40 right before mice rechallenged with the secondary tumours; **d**, **e** Proportions of CD44^+^CD62L^−^TEM and CD44^+^CD62L^+^TCM among CD4^+^ and CD8^+^ T cells. (n = 3). **f**–**h** Cytokine levels in sera from mice isolated at day 40 right before rechallenging mice with secondary tumors. (n = 3) Error bars indicate s.e.m. Statistical significance was calculated by one-way ANOVA Tukey's multiple comparisons test. *P*-value: *, *P* < 0.05; **, *P* < 0.01; ***, *P* < 0.001

Immune-mediated memory responses can protect against antigens that have been previously encountered through the rapid induction of recall responses [44]. CTLA-4 blockade can enhance the expansion and effector activity of memory CD8^+^T cells [45]. To assess the immunological memory induction following combination SDT + Zr-TCPP(TPP)/R837@M NP treatment, we subjected mice in which primary tumors had been eliminated to tumor rechallenge on day 40 post-surgery. Animals were then separated into four treatment groups, with five mice per group: (1) surgery; (2) surgery + anti-CTLA-4; (3) surgery + Zr-TCPP(TPP)/R837@M + US + anti-CTLA-4; and (4) surgery + Zr-TCPP/R837@M + US + anti-CTLA-4. Appropriate mice were treated with anti-CTLA-4 (10 μg/mouse/injection) on days 7, 9, and 11 following initial tumor removal (Fig. 6a). Images of 4T1 tumor-bearing mice were obtained since the second tumor inculation. (Additional file 1: Figure S7a) In animals that had undergone surgical primary tumor removal, secondary tumor growth was not significantly inhibited by anti-CTLA-4 treatment. However, for mice in the Zr-TCPP(TPP)/R837@M + US + anti-CTLA-4 treatment group, these secondary tumors grew very slowly, while in the photograph and the weight of excised rechallenged tumors in the Zr-TCPP/R837@M + US + anti-CTLA-4 treatment group were evidently smaller than other groups (Fig. 6b; Additional file 1: Figure S8b, c). These results thus indicated that SDT + Zr-TCPP(TPP)/R837@M + anti-CTLA-4 combination therapy was conducive to the establishment of durable antitumor immunity.

To assess immunological memory in this 4T1 mouse model system, lymph nodes were collected on day 40 prior to tumor rechallenge to assess the central memory and effector T cell (TCM and TEM, respectively) populations present therein. Memory T cells are able to mediate specific and efficient responses to pathogens or other sources of target antigens as they are present at high numbers, undergo persistent homeostatic population maintenance that is antigen-independent, and can rapidly expand when they encounter their cognate antigen. While TCMs begin rapidly expanding and differentiating following initial antigen stimulation, TEMs exhibit more robust and efficient acquisition of effector functions including cytokine production and cytolytic activity relative to TCMs [46, 47].

In both the CD4^+^ or CD8^+^ compartments, the frequencies of CD62L^−^CD44^+^ TEMs were significantly increased following Zr-TCPP(TPP)/R837@M-based SDT + anti-CTLA-4 treatment as compared to those frequencies observed in mice that underwent surgery with or without anti-CTLA-4 treatment (Fig. 6c–e). On day 40, serum cytokine levels in mice in these different groups were assessed via ELISA, revealing significant increases in the innate immunity-related IL-12p70 and the adaptive immunity-related TNF-α and IL-6 at this time point for mice that underwent combination Zr-TCPP(TPP)/R837@M-based SDT + anti-CTLA-4 treatment relative to the levels observed in mice that underwent surgery with or without CTLA-4 blockage. Relatively low expression levels were also evident when using NPs lacking mitochondria targeting activity (Fig. 6f–h). As such, combining Zr-TCPP(TPP)/R837@M and anti-CTLA-4 treatment can robustly enhance immunological memory to specifically prevent tumor recurrence.

## Conclusions

In conclusion, we herein developed a homologous mitochondria-targeting SDT platform that, when deployed in combination with CTLA-4 checkpoint blockade, was able to efficiently mediate antitumor immunotherapy. This platform was based on a combination of multifunctional nMOFs (Zr-TCPP(TPP)/R837@M) exhibiting efficient sonosensitizer loading and immune adjuvant activity, inducing ICD to promote TAA release while delivering R837 and thereby promoting DC maturation. By synergizing with anti-CTLA-4, this platform suppressed tumor progression, prevented metastatic development, and promoted the development of immunological memory. Together, these results highlight a novel approach to achieving noninvasive SDT to facilitate target tumor clearance.

## Materials and methods

### Materials

H_2_TCPP (379077), ZrOCl_2_·8H_2_O (224316), triphenylphosphonium (TPP, 157945), N, N-dimethylformamide (DMF, 227056), and Benzoic acid (242381) were from Sigma-Aldrich (St Louis, MO). (3-Carboxypropyl)triphenylphosphonium bromide (TPP, D051213) was from energy chemical. Imiquimod (R837, CAS No.: 99011-02-6) was from MCE. Anti-CTLA-4 (BE0164, USA) was from Bioxcell. Antibodies utilized for flow cytometry were from Biolegend.

### Cell culture

Murine 4T1 and 4T1-Luci cancer cell lines were from the American Type Culture Collection (ATCC) and were grown in RPMI-1640 (Gibco, NY, USA) supplemented with 10% fetal bovine serum (FBS) (Gibco, USA) and penicillin/streptomycin (Beyotime, Shanghai, China). Cells were cultured in a 37 °C humidified 5% CO_2_ incubator. Cell viability was monitored based on Trypan blue exclusion.

### 4T1 cell membrane isolation

A Membrane Protein Extraction Kit (Beyotime, Shanghai, China) was used to prepare 4T1 cell membranes. Briefly, 1 mL of membrane protein extraction buffer solution supplemented with PMSF (1 mM) was used to suspend 5 × 10^7^ 4T1 cells, which were then incubated for 10–15 min in an ice bath before being frozen to − 80 °C and then thawed to room temperature. The freeze–thaw process was repeated three times, after which samples were centrifuged for 15 min at 3500 rpm at 4 °C. Supernatants were then centrifuged for 30 min at 14,000 rpm at 4 °C, with precipitates being collected and stored at − 80 °C.

### Zr-TCPP(TPP)/R837@M nanoparticle synthesis

Initially, H_2_ TCPP (100 mg, 0.13 mmol), ZrOCl_2_·8H_2_O (300 mg, 0.93 mmol), and benzoic acid (2.9 g, 24 mmol) were dissolved in 100 mL of DMF and continuously stirred (300 rpm) for 5 h at 90 °C. Nanoparticles (NPs) were then collected by centrifuging this mixture for 10 min at 10,000 rpm, after which they were washed three times in DMF. Next, Zr-TCPP (75 mg) was suspended in methanol (20 mL), followed by sonication for 5 min. In addition, 4-carboxybutyl-TPP (35 mg) was dissolved in MeOH (20 mL), with the mixture then being supplemented with triethylamine (20 mL). These solutions were then combined together overnight at room temperature with constant stirring. NPs were then isolated via centrifugation, rinsed two times with methanol to remove free TPP, and dried overnight at room temperature under vacuum.

R837 loading was achieved via physical absorption. Briefly, R837 was loaded into the mitochondria-targeting MOF probe to yield Zr-TCPP(TPP)/R837, with the loading of these particles then being assessed via ultraviolet–visible (UV–Vis) spectrophotometry.

Zr-TCPP(TPP)/R837@M construction was achieved using a mini extruder (Avanti Polar Lipids). Briefly, the 4T1 cell membranes and the Zr-TCPP(TPP)/R837 were dissolved at 2 mg/mL in distilled water, followed by sonication for 15 min. The mini extruder was then used to extrude these cell membranes a minimum of 10 ties using a polycarbonate porous membrane (400 nm). Subsequently, 0.5 mL of Zr-TCPP(TPP)/R837 (2 mg/mL) was added to pretreated 4T1 cell membranes, with the solution again being extruded a minimum of 10 times, this time using a smaller polycarbonate porous membrane (200 nm). After extrusion, samples were collected via centrifugation for 10 min at 6000 rpm at 4 °C, after which the free membrane was removed by washing three times using water.

### Nanoparticle characterization

Transmission electron microscopy (TEM) and high-resolution TEM (HR-TEM) were utilized to assess Zr-TCPP(TPP)/R837@M structural characteristics. X-ray diffractometry was used to assess NP structural performance. The elemental composition and valence states of Zr-TCPP(TPP)/R837@M were verified via X-ray photoelectron spectroscopy (XPS). Zr-TCPP(TPP)/R837 and Zr-TCPP(TPP)/R837@M zeta potential and dynamic light scattering (DLS) characteristics were assessed with a particle analyzer (Nano-ZS, Malvern, England) at room temperature. A UV–vis spectrophotometer (Tecan Spark, Tecan, Switzerland)was used to assess Zr-TCPP(TPP) @M, R837, and Zr-TCPP(TPP)/R837@M UV–vis absorption spectra. Zr-TCPP(TPP)/R837@M NP crystal structure properties were established through X-ray powder diffraction analyses.

Cell membrane proteins were characterized via 12% SDS-PAGE separation followed by staining with Coomassie Brilliant Blue for 1 h and multiple rounds of destaining.

### Cellular uptake and efflux analyses

#### Assessment of Zr-TCPP(TPP)/R837@M subcellular localization

4T1 cells were incubated for 8 h with Zr-TCPP(TPP)/R837@M (50 mg/L), after which cells were rinsed using PBS and stained with MitoTracker Green (M7514, Invitrogen, USA) and Hoechst 33342 (Invitrogen, H1398, US) prior to fluorescent analysis via CLSM.

#### Cellular uptake assays

Zr-TCPP(TPP)/R837@M NP uptake was measured via flow cytometry (Beckman CytoFLEX; Beckman Coulter, Inc.). Briefly, 4T1 cells (3 × 10^5^/well in 2 mL) were added to 6-well plates for 24 h, after which media was exchanged for media supplemented with Zr-TCPP(TPP)/R837@M (20 µg/mL). Following incubation for 4, 8, 12, or 24 h, cells were rinsed using PBS and assessed via flow cytometry (EX = 405 nm, EM = 780 nm).

#### Homologous targeting analyses

Four different tumor cell types (4T1, MDA-MB-468, Hepa1-6, and Bxpc-3) were plated at 2 × 10^4^/well in 200 μL of media in 6-well chambered coverglass (Corning, USA). Zr-TCPP(TPP)/R837 and Zr-TCPP(TPP)/R837@M were then incubated with these cell lines for 8 h, after which they were harvested using trypsin, digested twice with PBS, and examined via flow cytometry.

### Evaluation of the ^1^O_2_ Generation Ability of Zr-TCPP(TPP)/R837@M

A fluorescence spectrometer and CLSM were utilized to assess ^1^O_2_ generation via a singlet oxygen sensor green (SOSG) assay (MA0326, Meilune, China) and DCFH-DA (Meilune, MB4682, China), respectively. For fluorescence spectrometry, Zr-TCPP(TPP)/R837@M was combined with SOSG (5 μM) in PBS, and the resultant SOSG + Zr-TCPP(TPP)/R837@M preparation (0.1 mg/mL) was sonicated under US irradiation (1 MHz, 1 W/cm^2^, duty cycle 50%, Sonicator 740, Mettler Electronics, USA) for a range of time periods (0, 10 s, 30 s, 1 min, 2 min, and 3 min). SOSG fluorescence was then measured at respective excitation and emission wavelengths of 504 and 525 nm (Tecan Spark, Tecan, Switzerland).

In CLSM analyses, 4T1 cells were treated for 8 h with Zr-TCPP(TPP)/R837@M (50 mg/L), followed by the addition of DCFH-DA (200 µL, 10 µM). Cells were then incubated for 30 min, washed three times with PBS, and subjected to US stimulation (1 MHz, 1 W/cm^2^, duty cycle 50%, 1 min). After being fixed for 15 min in 4% paraformaldehyde at room temperature, cells were washed thrice with PBS, stained for 5 min with DAPI, and washed three more times with PBS. A CLSM (Leica Microsystems, Wetzlar, Germany) was then employed to measure ROS generation.

### Evaluation of ICD induction

CRT exposure was measured by immunofluorescence staining and flow cytometry. Briefly, 4T1 cells were cultured with different treatments for 24 h, incubated with anti-Calreticulin antibody (ab92516, Abcam), 1,1′-dioctadecyl-3,3,3′,3′-tetramethylindocarbocyanine perchlorate (Dil, C1036, Beyotime) and Alexa Fluor^®^ 488 Conjugate (4412S, CST), then tested by CLSM and flow cytometry. HMGB1 release was accessed through immunofluorescence staining and ELISA. 4T1 cells were cultured with different treatments for 48 h, incubated with anti-HMGB1 antibody (ab18256, Abcam), Alexa Fluor^®^ 488 Conjugate (4412S, CST), and DAPI Staining Solution (C1006, Beyotime), then tested by CLSM, the cell supernatants were collected for HMGB1 release (ARG81351, arigo) and ATP release (S0027, Beyotime).

### In vitro cytotoxicity assay

Cellular cytotoxicity was assessed via CCK-8 assay (BS350B, Biosharp). Briefly, 4T1 cells were added to 96-well plates for 12 h, followed by treatment for 24 h with Zr-TCPP(TPP)/R837@M at a range of doses (0, 12.5, 25, 50, 100, 200 μg/mL). CCK-8 assays were then conducted, with absorbance being assessed at 450 nm via microplate reader (Tecan, Switzerland).

### Tumor targeting and tissue distribution

Female Balb/c mice (Shanghai SLAC Laboratory Animal Co, Ltd.) were utilized in all animal studies, which were approved by the Animal Laboratory of the Second Affiliated Hospital of Zhejiang University (permit no. 2021-118). A murine tumor model was established by subcutaneously implanting 4T1 cells in the breast pads of these mice when they were 6–8 weeks old, followed by intravenous treatment with 200 μL of Zr-TCPP(TPP)/R837@M or Zr-TCPP(TPP)/R837 (5 mg/kg) as appropriate. Tissue distributions in these animals were assessed by euthanizing them at appropriate time points and collecting tumors and major organs (heart, lungs, kidney, liver, spleen). An IVIS Spectrum imaging system (PerkinElmer, USA) was then utilized to conduct a real-time region of interest (ROI) fluorescence intensity analysis for Zr-TCPP(TPP)/R837@M at 0, 4, 8, 12, 20, 24, 36 and 48 h.

### Combination of SDT and ICB antitumor treatment

Primary model tumors were established by subcutaneously implanting 5 × 10^5^ 4T1 cells suspended in PBS into the right mammary fat pads of Balb/c mice, followed 6 days later by the implantation of the same number of tumor cells into the left mammary pads of these same animals to establish a bilateral tumor model. Tumor-bearing mice were then randomized into the following treatment groups: (1) PBS, (2) US, (3) Zr-TCPP(TPP) @M + US (4) Zr-TCPP(TPP) @M + US + anti- CTLA-4, (5) Zr-TCPP(TPP)/R837 @M + US, (6) Zr-TCPP(TPP) @M + US + anti- CTLA-4, and (7) Zr-TCPP@M + US + anti-CTLA-4. All US irradiation was performed at 24 h post-injection using identical treatment settings (3 MHz, 1.5 W/cm^2^, duty cycle 50%, Sonicator 740, Mettler Electronics, USA). For combination ICB treatment, mice were injected with anti-CTLA-4 (50 μg/mouse) on days 1, 3, and 5. Tumor volumes were measured as follows: (width^2^ × length)/2. At the experimental endpoint, mice were euthanized and tumors were collected and imaged. Mice were additionally euthanized when tumors were larger than 1000 mm^3^ as per standard protocols.

An orthotopic 4T1 tumor model was established by implanting mice with 4T1-Luci cells (5 × 10^5^) in the mammary fat pad, as above, followed by intravenous tail vein administration of additional 4T1-Luci cells (1 × 10^5^) 6 days later. Seven days after incubation of the orthotopic tumor, orthotopic tumors were ~ 50 mm^3^ in size, primary tumors were subjected to surgical treatment with or without anti-CTLA-4 administration. An appropriate substrate was injected into mice before bioluminescent imaging was conducted with an IVIS spectrum system.

Immunological memory was assessed by subcutaneously implanting 1 × 10^6^ 4T1 cells in PBS into the right mammary fat pad of individual female Balb/c mice. After these primary tumors had grown to ~ 50 mm^3^, they were surgically removed. After 40 days, mice were implanted with a secondary tumor and were intravenously injected with anti-CTLA-4 at appropriate time points (50 μg/mouse).

### In vivo immune response analysis

A total of 30 orthotopic tumor-bearing mice were selected and randomly assigned to the control, R837, Zr-TCPP(TPP)/R837@M, Zr-TCPP@M + US, Zr-TCPP(TPP)/R837@M + US, and Zr-TCPP/R837@M + US groups. Following treatment, samples of tumors and tumor-draining lymph nodes (DLNs) were collected and homogenized to produce single-cell suspensions. Cells in blood and tumor samples were stained using the following reagents prior to flow cytometry analysis: Zombie NIR™ Fixable Viability Kit (423105, Biolegend, UK), Brilliant Violet 605™ anti-mouse CD45 (103139, Biolegend, UK), PE/Cyanine7 anti-mouse CD3 (100220, Biolegend, UK), Brilliant Violet 421™ anti-mouse CD4 (100438, Biolegend, UK), Alexa Fluor^®^ 647 anti-mouse Foxp3 (126408, Biolegend, UK).

Cells collected from DLNs were stained with the following reagents prior to flow cytometry analysis: Zombie Aqua™ Fixable Viability Kit (423101, Biolegend, UK), Brilliant Violet 421™ anti-mouse CD11c (117329, Biolegend, UK), PE/Cyanine7 anti-mouse CD80 (104733, Biolegend, UK), Brilliant Violet 785™ anti-mouse CD86 (105043, Biolegend, UK).

Immune cell infiltration in secondary tumors was assessed by collecting these cells, preparing a single-cell suspension as above, and staining them with the following reagents prior to flow cytometry analysis: Zombie NIR™ Fixable Viability Kit (423105, Biolegend, UK), Brilliant Violet 605™ anti-mouse CD45 (103139, Biolegend, UK), PE/Cyanine7 anti-mouse CD3 (100220, Biolegend, UK), Brilliant Violet 421™ anti-mouse CD4 (100438, Biolegend, UK), Brilliant Violet 785™ anti-mouse CD8a (100750, Biolegend, UK), Alexa Fluor^®^ 647 anti-mouse Foxp3 (126408, Biolegend, UK).

Memory T cells were analyzed in single-cell suspensions prepared from homogenized lymph nodes stained with the following reagents prior to flow cytometry analysis: Zombie NIR™ Fixable Viability Kit (423105, Biolegend, UK), PE/Cyanine7 anti-mouse CD3 (100220, Biolegend, UK), Brilliant Violet 421™ anti-mouse CD4 (100438, Biolegend, UK), Brilliant Violet 510™ anti-mouse CD8a (100752, Biolegend, UK), APC anti-mouse CD62L (104412, Biolegend, UK) and PE anti-mouse/human CD44 (103008, Biolegend, UK). Central memory and effector memory T cells (TCM and TEM) exhibited respective CD3^+^CD8^+^CD62L^+^CD44^+^ and CD3^+^CD8^+^CD62L^−^CD44^+^ staining profiles. All antibodies used for these staining assays were diluted 1:100.

### Cytokine detection

Samples of murine serum were collected and diluted to appropriate concentrations. ELISA kits were used to measure levels of tumor necrosis factor (SEKM-0034, Solarbio), IL-12p40 (SEKM-0012, Solarbio), and IL-6 (1210602, Dakewe biotech) in these samples.

### Statistical analysis

Data are means ± standard deviation and were assessed using one-way ANOVAs with Tukey’s post-hoc correction for multiple testing or Dunnett’s multiple comparisons test. ns: *P* > 0.05, *P < 0.05, **P < 0.01, ***P < 0.001 and ****P < 0.0001.

## Supplementary Information

**Additional file 1: Figure S1. a** Cellular uptake of Zr-TCPP(TPP)/R837@M. **b** In vitro cell viability of 4T1 cells. **Figure S2.** 4T1, MDA-MB-468, Hepa1-6, and Bxpc-3 after 8 h incubation with Zr-TCPP(TPP)/R837. **Figure S3.** CRT exposure. **Figure S4. a** The ex vivo fluorescence image of major organs and tumour and **b** quantification analysis of the tissue content of Zr-TCPP(TPP)/R837 and Zr-TCPP(TPP)/R837@M; data are expressed as means ± SD (n = 3). Statistical significance was calculated by one-way ANOVA Tukey's multiple comparisons test. *P*-value: *P < 0.05; **P < 0.01; ***P < 0.001. **Figure S5. a** Images of 4T1 tumor-bearing mice. **b** Proportions of tumor-infiltrating CD45^+^ and CD3^+^ T cells among distant tumor cells. (n = 3) Statistical significance was calculated by one-way ANOVA Dunnett’s multiple comparisons test. *P*-value: *P < 0.05; **P < 0.01; ***P < 0.001. **Figure S6.** Hemolysis of Zr-TCPP(TPP)/R837@M NPs. **Figure S7.** Images of 4T1 tumor-bearing mice over 18 d after different treatments and corresponding HE staining of major organs (heart, liver, spleen, lung, and kidney) of mice after various treatments. Scale bar = 20um. **Figure S8. a** Images of 4T1 tumor-bearing mice over 62 days after different treatments; **b** photographs of excised rechallenged tumors at the end of treatments; **c** average weight of rechallenged tumors at the end of treatments (n = 5) Statistical significance was calculated by one-way ANOVA Tukey's multiple comparisons test. *P*-value: *P < 0.05; **P < 0.01; ***P < 0.001.
