# Supplementary material for: Enhancement of antitumor immunotherapy using mitochondria-targeted cancer cell membrane-biomimetic MOF-mediated sonodynamic therapy and checkpoint blockade immunotherapy
Source: J Nanobiotechnology. 2022 May 14;20:228. doi: 10.1186/s12951-022-01453-2 (PMC9107704; doi:10.1186/s12951-022-01453-2)
Supplement: Supplementary file 1 — Additional file 1: Figure S1. a Cellular uptake of Zr-TCPP(TPP)/R837@M. b In vitro cell viability of 4T1 cells. Figure S2. 4T1, MDA-MB-468, Hepa1-6, and Bxpc-3 after 8 h incubation with Zr-TCPP(TPP)/R837. Figure S3. CRT exposure. Figure S4. a The ex vivo fluorescence image of major organs and tumour and b quantification analysis of the tissue content of Zr-TCPP(TPP)/R837 and Zr-TCPP(TPP)/R837@M; data are expressed as means ± SD (n = 3). Statistical significance was calculated by one-way ANOVA Tukey's multiple comparisons test. P-value: *P < 0.05; **P < 0.01; ***P < 0.001. Figure S5. a Images of 4T1 tumor-bearing mice. b Proportions of tumor-infiltrating CD45+ and CD3+ T cells among distant tumor cells. (n = 3) Statistical significance was calculated by one-way ANOVA Dunnett’s multiple comparisons test. P-value: *P < 0.05; **P < 0.01; ***P < 0.001. Figure S6. Hemolysis of Zr-TCPP(TPP)/R837@M NPs. Figure S7. Images of 4T1 tumor-bearing mice over 18 d after different treatments and corresponding HE staining of major organs (heart, liver, spleen, lung, and kidney) of mice after various treatments. Scale bar = 20um. Figure S8. a Images of 4T1 tumor-bearing mice over 62 days after different treatments; b photographs of excised rechallenged tumors at the end of treatments; c average weight of rechallenged tumors at the end of treatments (n = 5) Statistical significance was calculated by one-way ANOVA Tukey's multiple comparisons test. P-value: *P < 0.05; **P < 0.01; ***P < 0.001. [file 12951_2022_1453_MOESM1_ESM.docx]

**Supplementary Information**

Enhancement of antitumor immunotherapy using mitochondria-targeted cancer cell membrane-biomimetic MOF-mediated sonodynamic therapy and checkpoint blockade immunotherapy

Jiali Luo^1,2,‡^, Xue Wang^1,2,‡^, Zhan Shi^1,2^, Yiqing Zeng^1,2^, Liangcan He^3^, Jing Cao^1,2^, Yu Sun^1,2^, Tao Zhang^1,2,*^, and Pintong Huang^1,2,*^

^1^Department of Ultrasound in Medicine, The Second Affiliated Hospital of Zhejiang University School of Medicine, No.88 Jiefang Road, Shangcheng District, Hangzhou 310009, P.R. China

^2^Research Center of Ultrasound in Medicine and Biomedical Engineering, The Second Affiliated Hospital of Zhejiang University School of Medicine, No.88 Jiefang Road, Zhejiang University, Shangcheng District, Hangzhou 310009, P.R. China.

^3^School of Medicine and Health, Harbin Institute of Technology, Harbin 150080, P.R. China

Supplementary Figures:


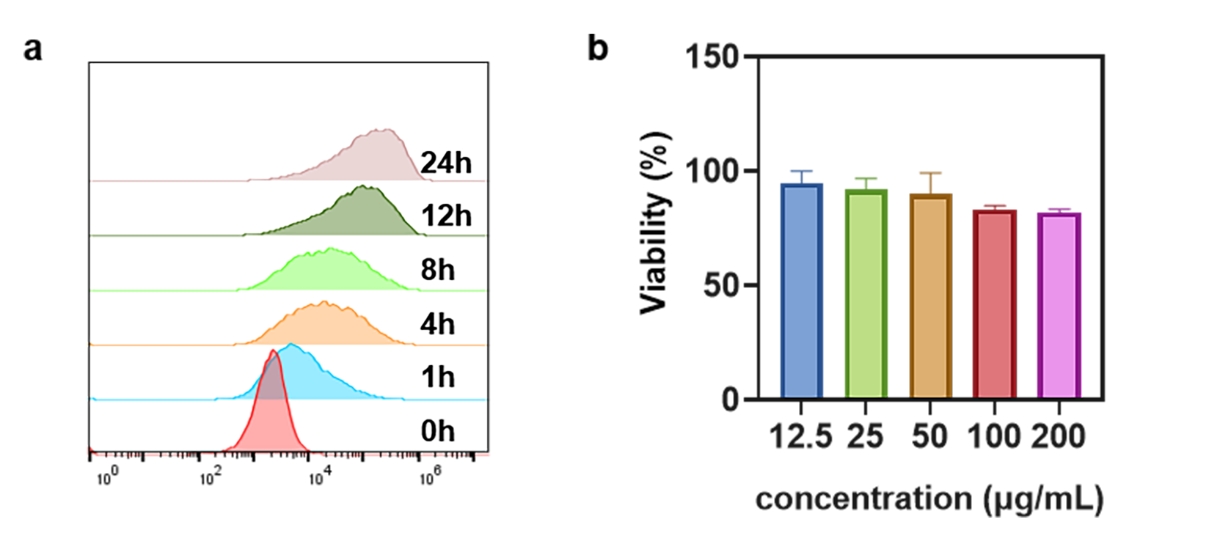


**Figure S1** (a) Cellular uptake of Zr-TCPP(TPP)/R837@M by flow cytometry at different time points. (b) *In vitro* cell viability of 4T1 cells after treatment with various concentrations of Zr-TCPP(TPP)/R837@M NPs.


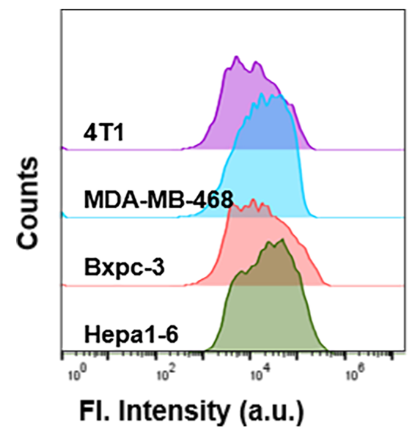


**Figure S2** 4T1, MDA-MB-468, Hepa1-6, and Bxpc-3 after 8h incubation with Zr-TCPP(TPP)/R837 were examined by flow cytometry.


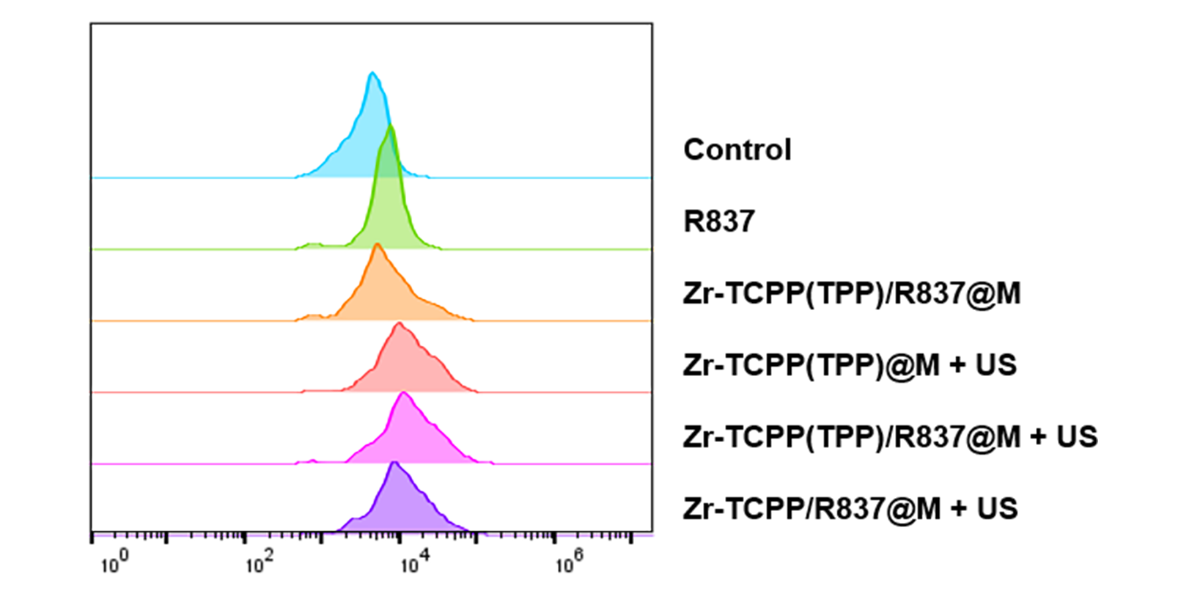


**Figure S3** CRT exposure after different treatments measured by flow-cytometry.


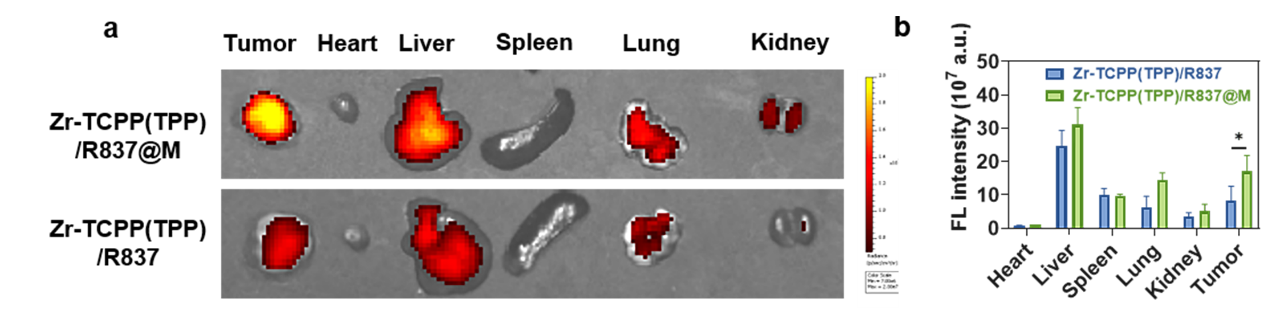


**Figure S4** (a) The *ex vivo* fluorescence image of major organs and tumor dissected from the mouse 24 h post injection and (b) Quantification analysis of the tissue content of Zr-TCPP(TPP)/R837 and Zr-TCPP(TPP)/R837@M by testing the corresponding fluorescence intensity; data are expressed as means ± SD (n = 3). Statistical significance was calculated by one-way ANOVA Tukey's multiple comparisons test. *P*-value: *, *P* < 0.05; **, *P* < 0.01; ***, *P* < 0.001

**
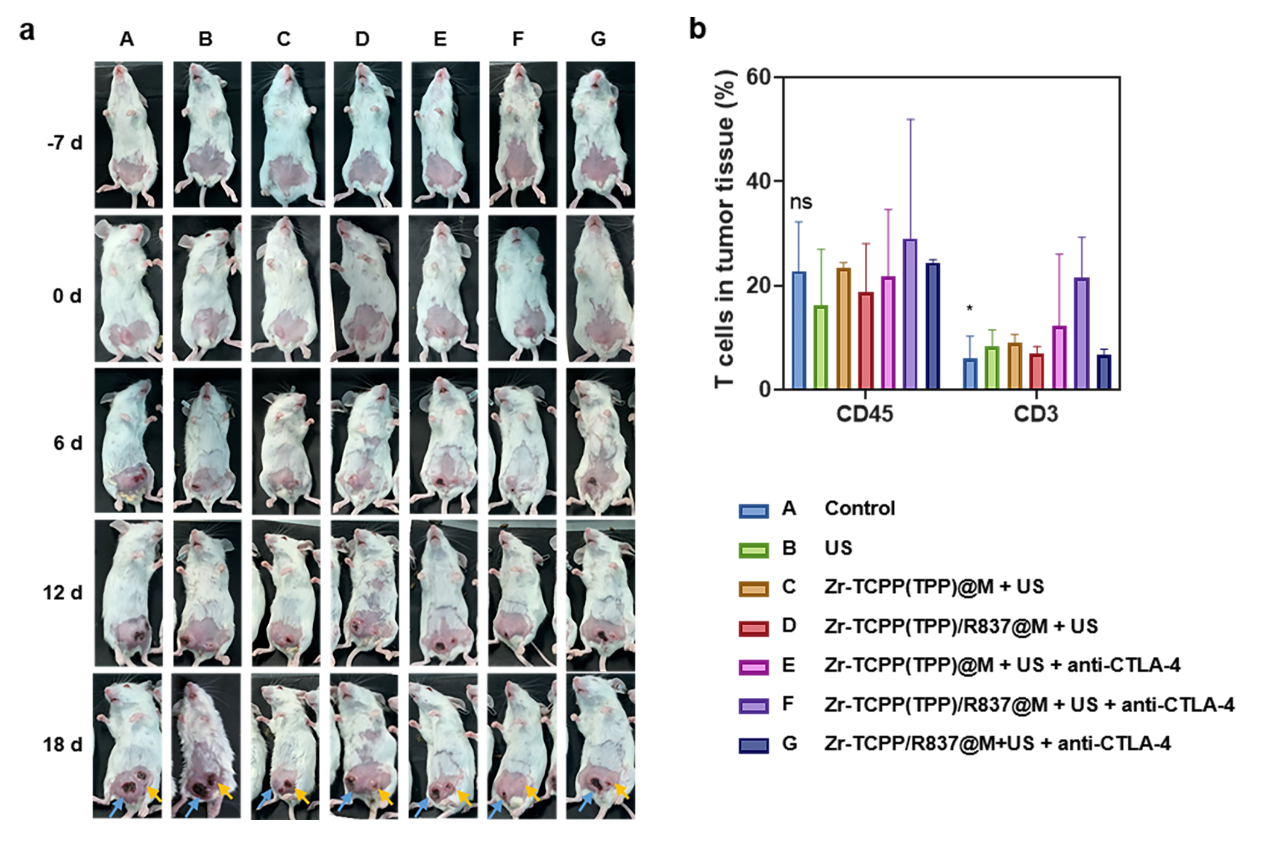
Figure S5** (a) Images of 4T1 tumor-bearing mice over 18 d after different treatments. (b) Proportions of tumor-infiltrating CD45^+^ and CD3^+^ T cells among distant tumor cells. (n = 3) Statistical significance was calculated by one-way ANOVA Dunnett's multiple comparisons test. *P*-value: *, *P* < 0.05; **, *P* < 0.01; ***, *P* < 0.001.


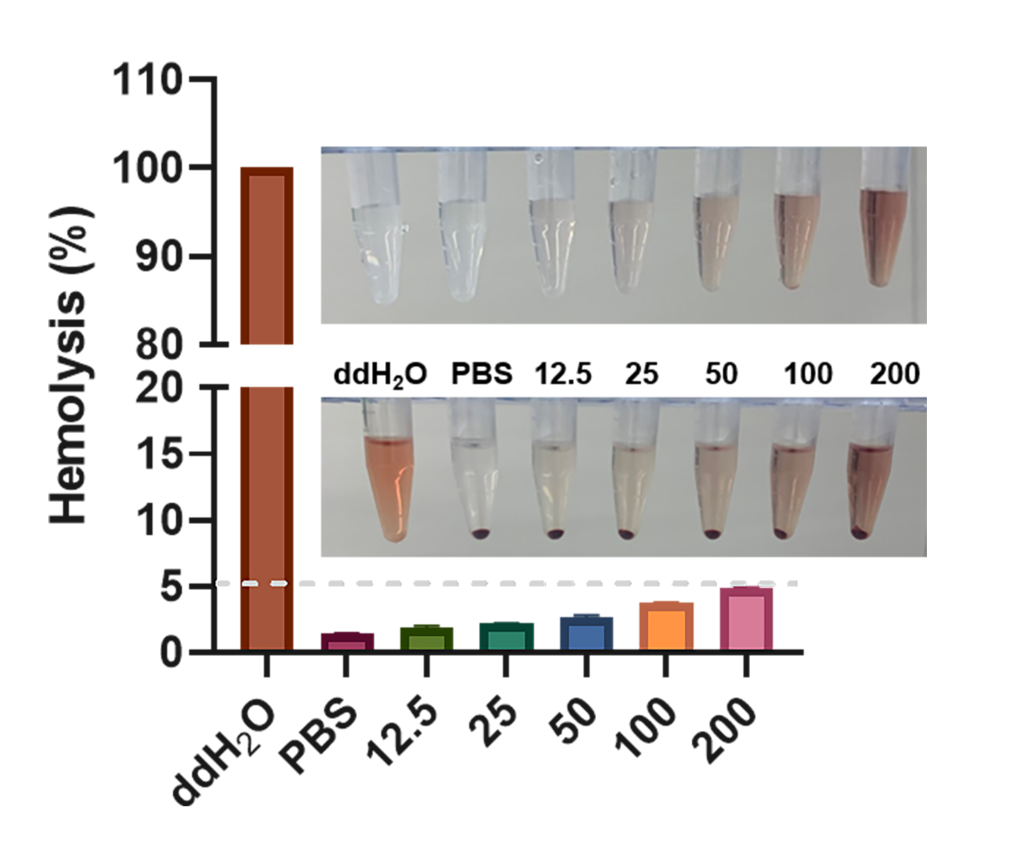


**Figure S6** Hemolysis of Zr-TCPP(TPP)/R837@M NPs after incubation with red blood cells with various concentrations, PBS as a negative and deionized water as a positive control respectively. Inset: hemolysis photographs after centrifugation.


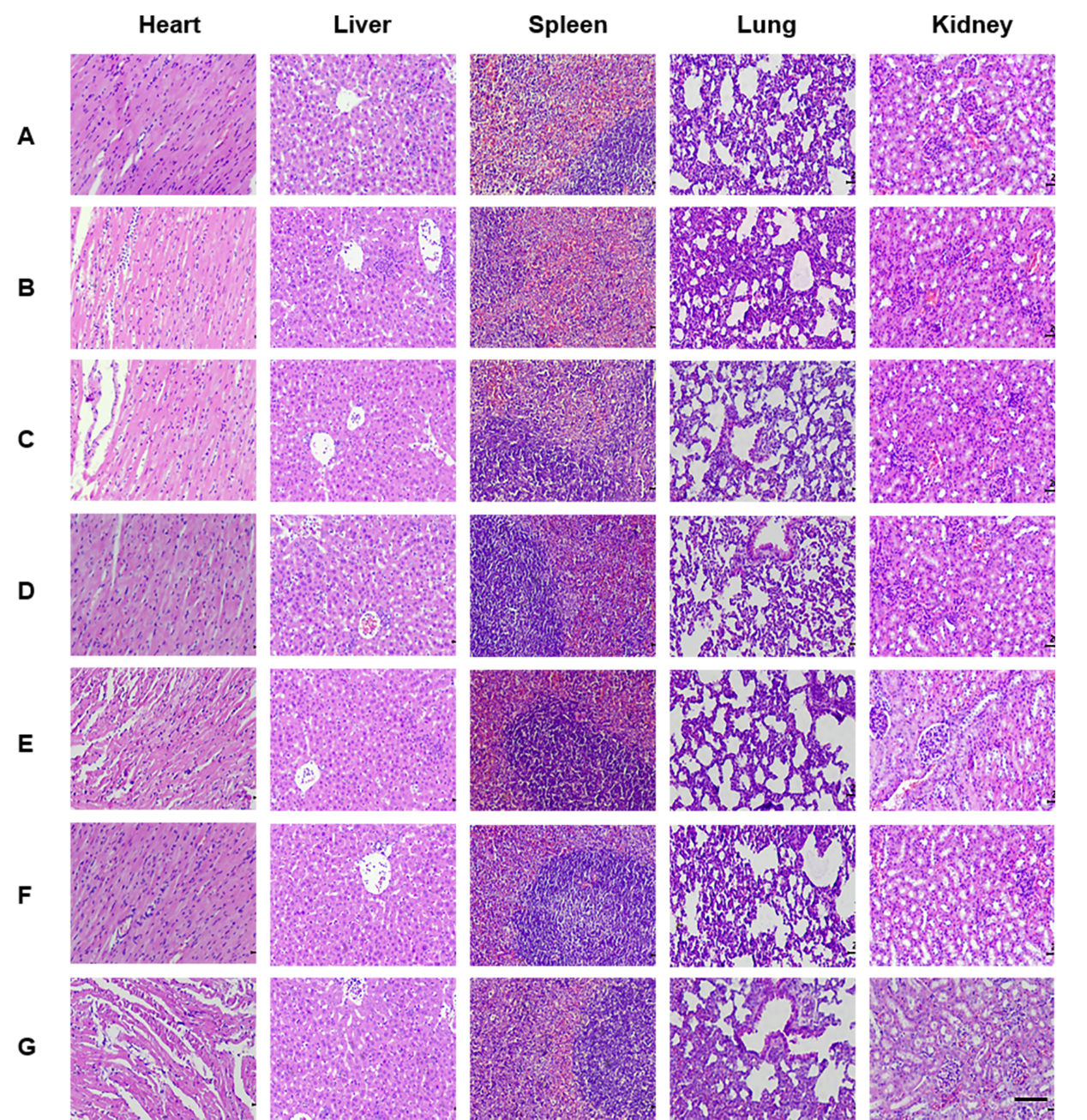


**Figure S7** Images of 4T1 tumor-bearing mice over 18 d after different treatments and corresponding HE staining of major organs (heart, liver, spleen, lung and kidney) of mice after various treatments. Scale bar = 20um. A Control; B US; C Zr-TCPP(TPP)@M + US; D Zr-TCPP(TPP)/R837@M + US; E Zr-TCPP(TPP)@M + US + anti-CTLA-4; F Zr-TCPP(TPP)/R837@M + US + anti-CTLA-4; G [Zr-TCPP/R837@M + US + anti-CTLA-4.](mailto:Zr-TCPP/R837@M+US+anti-CTLA-4.)


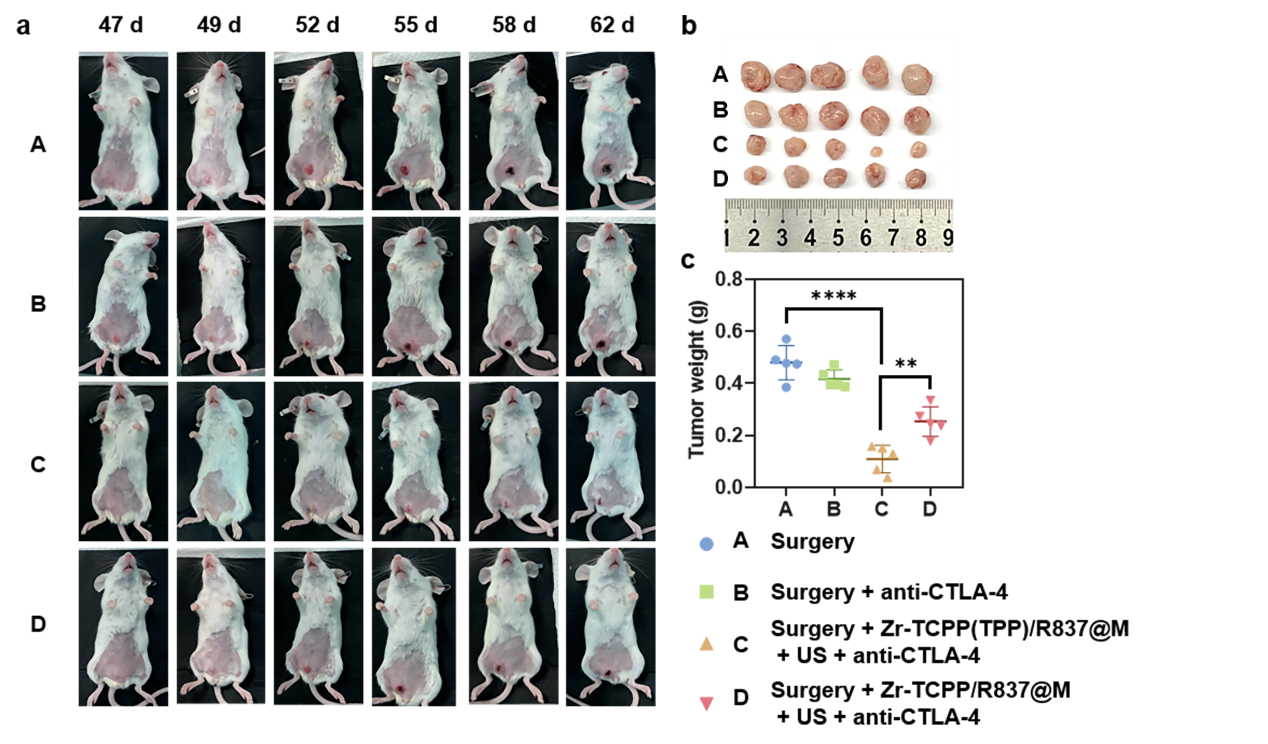


**Figure S8** (a) Images of 4T1 tumor-bearing mice over 62 days after different treatments; (b) Photographs of excised rechallenged tumors at the end of treatments; (c) Average weight of rechallenged tumors at the end of treatments. (n = 5) Statistical significance was calculated by one-way ANOVA Tukey's multiple comparisons test. *P*-value: *, *P* < 0.05; **, *P* < 0.01; ***, *P* < 0.001.
